# Necrotizing *E. coli* pneumonia with subsequent pneumothorax in a dog: a case report

**DOI:** 10.3389/fvets.2025.1587810

**Published:** 2025-09-18

**Authors:** Victoria Vida Vazin, Sara Huebner, Teagan Deforge, Michael Wood, Athanasia Mitropoulou

**Affiliations:** ^1^Department of Medical Sciences, School of Veterinary Medicine, University of Wisconsin-Madison, Madison, WI, United States; ^2^Department of Clinical Sciences, School of Veterinary Medicine, Louisiana State University, Baton Rouge, LA, United States

**Keywords:** necrotizing, pneumonia, dog, *Escherichia coli*, pneumothorax, aspiration

## Abstract

Necrotizing pneumonia is a poorly described and rarely reported phenomenon in veterinary medicine. The objective of this case report is to describe the successful management of a case of necrotizing pneumonia in a dog requiring medical and surgical management. This case describes a 9-year-old male neutered Siberian Husky mix that presented to an emergency center for a chronic cough not responsive to antibiotics including doxycycline and amoxicillin—clavulanate. Diagnostic imaging studies were consistent with severe multilobular pneumonia, pleural effusion and secondary pneumothorax. Aerobic culture of a bronchoalveolar lavage sample isolated *Escherichia coli* that was susceptible to enrofloxacin. A median sternotomy was performed when antibiotics alone were unable to fully clear the infection and the cranial segment of the left cranial lung lobe was removed. Histopathology of this lung lobe indicated subacute suppurative and fibrinohemorrhagic bronchopneumonia with parenchymal and pleural necrosis. The dog survived to discharge with resolution of pneumonia based on thoracic radiographs after 38 days. Necrotizing pneumonia can potentially be adequately treated with appropriate escalation of medical and surgical management.

## Introduction

Necrotizing pneumonia is an uncommon severe complication of bacterial community acquired pneumonia (CAP) in humans and an uncommon phenomenon due to any strain of bacteria in veterinary medicine (1). It is a consequence of severe inflammation affecting a single lung lobe or cluster of alveoli characterized by pulmonary inflammation with consolidation, multiple small cavities, and peripheral necrosis (1). In humans, *Staphylococcus aureus* and *Streptococcus* spp. are the most common causative agents, however, there are many other documented infectious agents including *Blastomycosis dermatitidis, Mycoplasma pneumoniae*, H1N1 influenza, *Pseudomonas aeruginosa*, *Klebsiella* spp., and *Escherichia coli* (2–5). Histopathologically, fibinohemorrhagic necrosis is the main finding within the lung parenchyma in combination with blood vessel necrosis, fibrin thrombi, neutrophilic and histiocytic infiltrates (6–8). There are few reports in veterinary medicine describing necrotizing pneumonia, both with and without recovery (6–9). Two case reports from research facilities reported that they performed necropsies on dogs that acutely died or were euthanized in their facilities from suspected pneumonia with findings indicating necrohemorrhagic pneumonia associated with *E. coli* strains as the causative factor (6). Additionally, there is a report of 13 cats with a history of acute respiratory clinical signs that acutely died in a shelter environment showing acute necrotizing pneumonia with *E. coli* isolates on necropsy without an apparent pneumothorax (7). The cause for the development of pneumonia was unclear. In limited veterinary case reports, the prognosis for dogs is poor with a single case report describing the survival of a dog with necrotizing pneumonia secondary to opportunistic bacterium *P. aeruginosa*. The development of pneumonia was likely secondary to devitalization of tissue from heat stroke and opportunistic bacteria colonization (9). In human medicine, the prognosis is considered good in children and guarded in adults with resolution of clinical signs and radiographic resolution in 5–6 months with antibiotic treatment and surgical intervention when indicated (1, 5). This case report describes a case of *E. coli* associated necrotizing pneumonia with subsequent pneumothorax that was successfully managed with medical and surgical management.

## Case report

A 9-year-old male neutered Siberian Husky mix weighing 35 kg was admitted to the emergency service at University of Wisconsin Veterinary Care. The owner reported that the dog had a 5–6 weeks history of a non-productive, intermittent hacking cough, intermittent lethargy and hyporexia. He had previously been treated with a 14-days course of doxycycline (8.5 mg/kg PO q12) and cough tablets (unspecified dose q8). Over 2 weeks, the owner noted initial mild improvement regarding appetite and energy, but the cough remained static with new development of serous nasal discharge. The cough became productive 2–3 weeks before presentation with production of mucoid material and the nasal discharge became mucoid. During this time, a 10-days course of amoxicillin—clavulanate (16 mg/kg PO q12) was prescribed with no improvement in clinical signs. Other pertinent history included chronic, intermittent (about once a week) regurgitation of food or liquid, usually after eating. This had not been investigated or treated. He does have a history of attending dog-daycare.

On presentation, the dog was alert, panting, with a rectal temperature of 105.6 F (40.8 °C). He had a mild amount of bilateral serous nasal discharge and soft crackles on auscultation of the left cranial hemithorax. The rest of the vital parameters were within the normal range. A point of care ultrasound of the thorax was performed as part of his triage examination. This showed diffuse B-lines and a shred sign of the left cranial lung lobe and occasional B-lines (1–2/field) of the right lung field. He was oxygenating at 95–96% on room air based on pulse oximetry. The patient was assessed to be stable on presentation.

**Figure 1:**
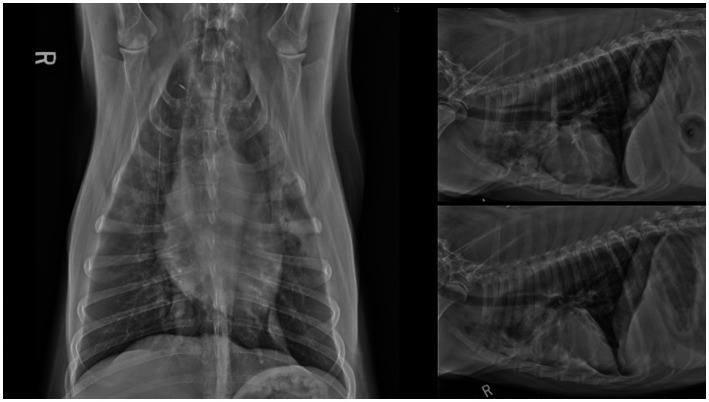
Ventrodorsal, right and left lateral thoracic radiographs performed on presentation. Ventrally distributed alveolar pulmonary pattern within left and right cranial lung lobes. Caudal intrathoracic esophageal soft tissue opacity.

**Figure 2:**
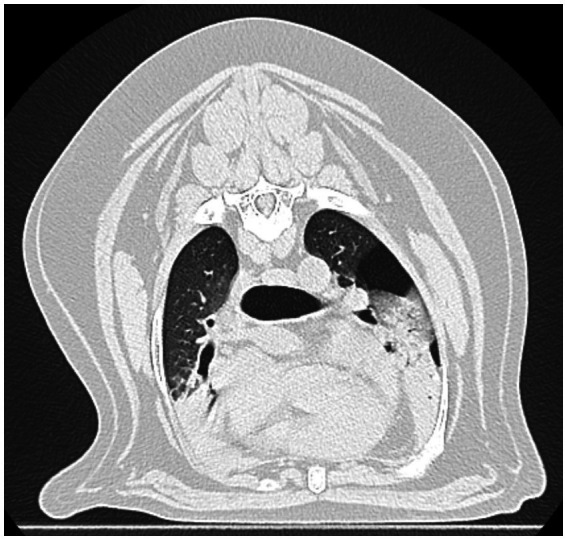
Computed tomography scan of thorax performed on first day of hospitalization. Ventrally distributed soft tissue attenuation throughout all lung lobes. Free gas in pleural space of the left hemithorax. Scant cranial mediastinal free gas.

Hematology on presentation showed a leukocytosis characterized by a regenerative left shift and mild toxic change. The serum biochemistry showed a minimally elevated alkaline phosphatase (ALP) (Table 1). A Blastomycosis urine antigen test was negative. Thoracic radiographs with three views showed multilobular pneumonia within the cranial and caudal subsegments of the left and right cranial lung lobe as well as retained esophageal fluid on the left lateral view (Figure 1). A computed tomography (CT) scan of the thorax (Figure 2) showed ventrally distributed soft tissue attenuation throughout all lung lobes, most severe in the left and right cranial lung lobes. There was moderate fluid filling of both cranial lobar bronchi and a moderate volume of free gas in the pleural space of the left hemithorax. Bronchoalveolar lavage fluid cytology samples consisted of many inflammatory cells including neutrophils (>80%), with a few macrophages, small lymphocytes, and rare eosinophil within large amounts of mucus. Bacteria were trapped in mucus and often phagocytosed by neutrophils. The interpretation of the cytology indicated severe neutrophilic inflammation with rode—shaped (bacilli) bacteria and minimal to moderate hemorrhage. Cultures from the bronchoalveolar lavage were performed that indicated heavy growth of *Escherichia coli* (*E. coli*) and no growth for *Mycoplasma*. Specific agents including *Nocardia* and *Actinomyces* were not specifically investigated. The dog’s initial antibiotic treatment was enrofloxacin (12 mg/kg IV q24) based on culture sensitivity (Table 2).

**Table 1:** Blood work values during hospitalization.

Parameter	Day 1	Day 4 (9 am)	Day 5 (9 am)	Day 6 (9 am)	Day 7 (8–10 am)	Reference range
WBC	20.5		23.3			5.0–14.0 × 10^3^/uL
Neutrophils	19.1		17.2			2.6–10.0 × 10^3^/uL
Bands	0.6		0.0			0.0–0.2 × 10^3^/uL
Lymphocytes	0.4		2.6			0.7–4.3 × 10^3^/uL
Monocytes	0.4		3.5			0.1–0.9 × 10^3^/uL
Eosinophils	0		0			× 10^3^/uL
Hematocrit	48		43			39–57%
RBC	7.51		7.4			5.60–8.4 × 10^6^/uL
Reticulocytes	0.029		0.009			0.013–0.102 × 10^6^/uL
PLT	342		315			175–500 × 10^3^/uL
Urea	8.0		5			7–32 mg/dL
Creatinine	0.9		0.7			0.5–1.5 mg/dL
Sodium (Na)	148.0	148.0	151.0	151.0	154.0	142–149 mmol/L
Potassium (K)	3.4	3.8	4.2	4.0	4.7	3.35–4.37 mmol/L
Ionized calcium (iCa)	1.30	1.34	1.32	1.30	1.35	1.23–1.43 mmol/L
Phosphorous	2.7		4.9			2.2–7.9 mg/dL
Albumin	3.4		2.7			2.3–3.9 g/dL
Globulin	3.5		3.7			3.5 g/dL
Total protein	6.8		6.5			2.2–3.5 g/dL
Glucose	80	119	97		87	67–132 mg/dL
Bilirubin	0.2		0.2			0.1–0.8 mg/dL
Cholesterol	311					149–319 mg/dL
Triglycerides	49					32–190 mg/dL
Alkaline phosphatase (ALP)	161					20–157 U/L
Alanine-amino-transferase (ALT)	46		42			14–87 U/L
Gamma-glutamyltransferase (g-GT)	<10					5–16 U/L
pH (venous)	7.42	7.331	7.38	7.221	7.37	7.35–7.45
pvCO_2_	27.6	30.9	39.9	57.3	31.0	35–45 mmHg
Bicarbonate	17.8	16.3	23.7	23.5	18.3	19–25 mmol/L
Base excess (BE)	−6.5	−9.5	−1.2	−4.1	−6.8	−5 to 5 mmol/L
Lactate	2.9	1.5	0.8	0.4	0.6	0.4–2.2 mmol/L

Blue indicates low values; red indicates elevated values; green indicates values within normal range; grey indicates absent values.

**Table 2:** Aerobic culture susceptibility results from bronchoalveolar lavage.

Antibiotic	Minimum inhibitory concentration (ug/mL)	Susceptibility
Ampicillin	≤2	Resistant
Amoxicillin/clavulanic acid	≤2	Resistant
Cefalexin	8	Intermediate
Cefpodoxime	≤0.25	Susceptible
Ceftazidime	≤0.12	Susceptible
Ceftiofur	≤1	Susceptible
Amikacin	≤2	Susceptible
Gentamicin	≤1	Susceptible
Doxycycline	≤0.5	Resistance
Ciprofloxacin	≤0.06	Susceptible
Enrofloxacin	≤0.12	Susceptible
Marbofloxacin	≤0.5	Susceptible
Trimethoprim/sulfamethoxazole	≤20	Susceptible
Chloramphenicol	≤2	Susceptible

On the fourth day of hospitalization, the dog became progressively tachypneic/dyspneic with a pulse oximeter reading 86% on room air and 97% on oxygen support. Nasal prongs were placed for flow-by oxygen support. Repeat thoracic radiographs were taken and showed a progressive pneumothorax compared to previous CT scan. Overnight, a thoracocentesis was performed that removed 1,380 mL and 580 mL of air from the left and right side of the thorax, respectively. A single lumen 18 Ga × 30 cm polyurethane chest tube with multiple fenestrations (MILA International, Florence, KY, USA) was aseptically placed on the left side of the thorax. A total of 1,120 mL air and scant free fluid was collected from the chest tube immediately after placement. Cytology of the fluid obtained from the chest tube directly after placement revealed an exudate characterized by marked neutrophilic inflammation with intracellular short rod-shaped (bacilli) bacteria. Although a culture was obtained at that time, it was not submitted. Due to progression of pneumothorax and evidence of a septic pyothorax with similar appearance to previous cytology of the bacteria, the dog underwent a thoracotomy for a lung lobectomy with the owner’s consent. It was decided to submit a new culture from tissue samples obtained during the thoracotomy.

**Figure 3:**
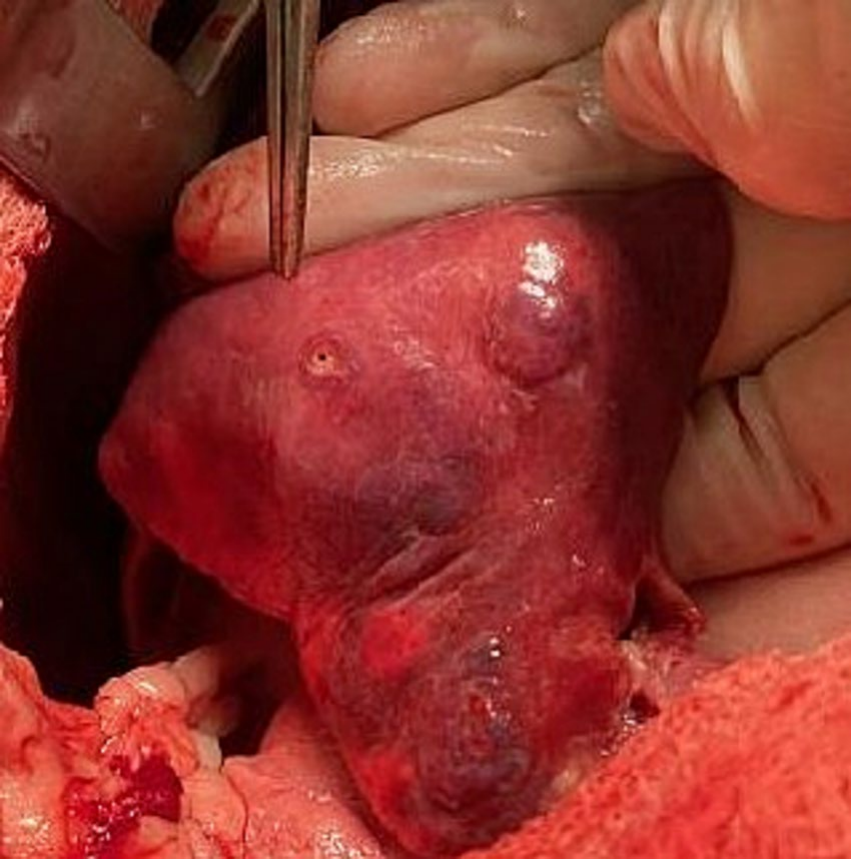
Picture of cranial subsegment of the left cranial lung lobe with a 0.9 × 1.7 cm defect.

The left cranial lung lobe was approached with a routine median sternotomy. A partial left cranial lung lobectomy of the cranial subsegment was performed. There was purulent material exuding from the bronchi. At the distal aspect of the lobe, there was a 0.9 × 1.7 cm defect in the pleura and parenchyma. The pneumothorax was attributed to a large focus of parenchymal and pleural necrosis at the distal aspect of the left cranial lung lobe. The remainder of the removed portion of the lung was firm, collapsed, and sank in the formalin jar. The distal 50% of the lobe was dark brown and the proximal 50% of the lobe was dark tan in color (Figure 3). An aerobic and anaerobic culture were obtained and submitted from the affected lung tissue after removal, with the remaining tissue submitted for histopathology. The dog was taken to the ICU for recovery and close monitoring.

**Figure 4:**
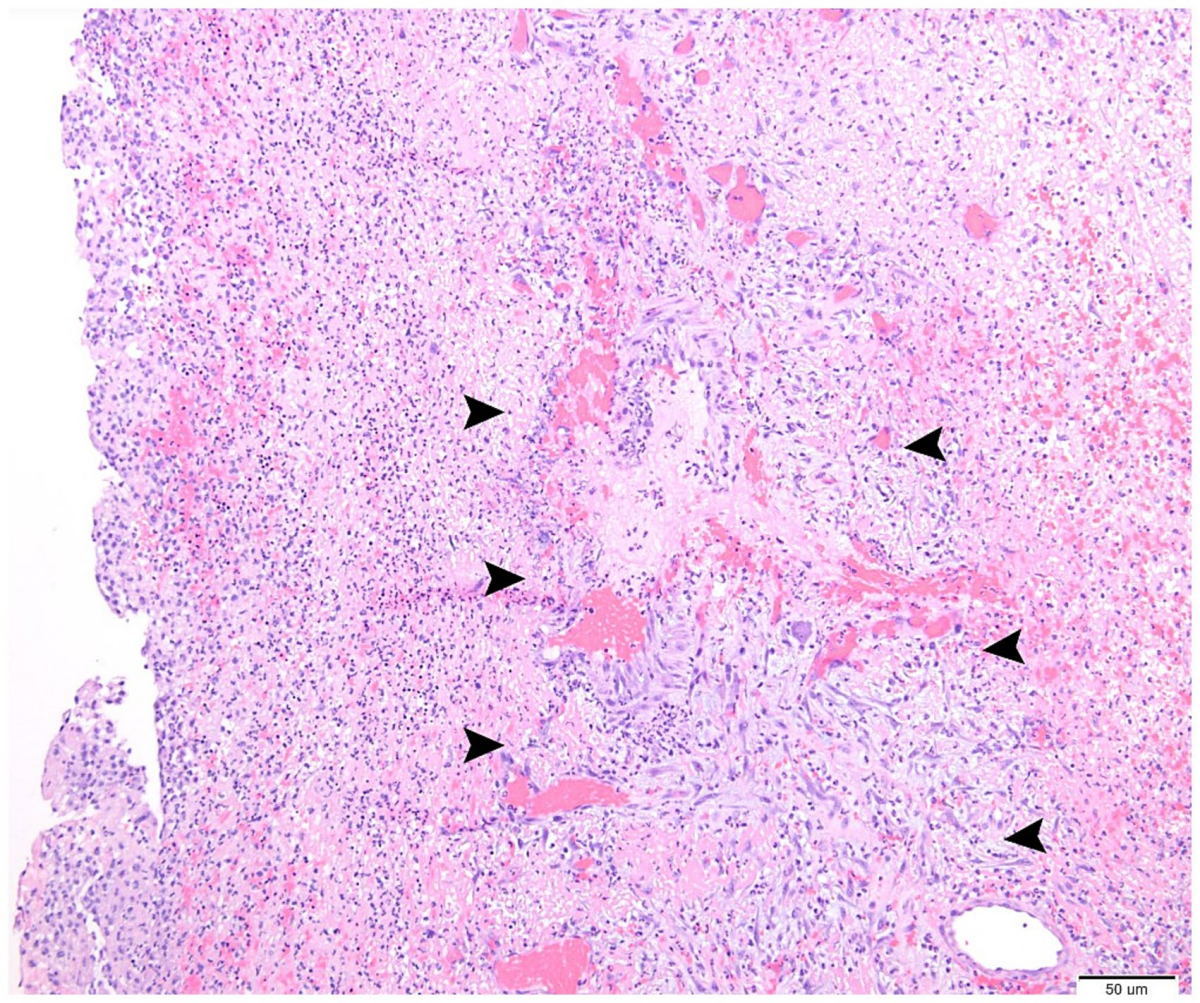
Large area of parenchymal and pleural necrosis with hemorrhage and fibrin aggregates, intact as well as fragmented neutrophils, and granulation tissue (arrowheads). Hematoxylin and eosin stain, 10 × objective (University of Madison, Madison, United States).

Histopathology indicated severe, lobar, subacute suppurative and fibinohemorrhagic bronchopneumonia and bronchitis with parenchymal and pleural necrosis (Figure 4). We attributed this pathology to bacterial infection with *E. coli*. In the more severely affected areas, there was parenchymal necrosis and interstitial inflammation suggestive of prior viral infection predisposing to secondary bacterial pneumonia. There was no growth after 7-days of the aerobic and anaerobic cultures of the lung tissue.

After surgery, flow-by oxygen supplementation was required to maintain the dogs SpO2 at 97%. The following medications were administered to the patient: plasmalyte constant rate infusion (CRI) (1.5 mL/kg/h), ondansetron (0.5 mg/kg PO q8 as needed) for 30 days, omeprazole (1 mg/kg IV q12 then switched to PO) for 35 days, visbiome (2 capsules PO q24) for 30 days, cisapride (0.2 mg/kg PO q8) for 35 days then increased dose (0.44 mg/kg PO q8) for 42 days, capromorelin (3 mg/kg PO q24 as needed) for 35 days, pregabalin (3 mg/kg PO q12) for 30 days, enrofloxacin (12 mg/kg PO q24) for 44 days, fentanyl patch (100 mcg/h) for 3 days, and saline nebulization with coupage for 5–10 min q6 for 3 days.

The dog made a good recovery over the next few days postoperatively. On day two after surgery, he was taken off supplemental oxygen and his measured SpO2 was 100% while breathing room air. His respiratory rate and effort were normal at this time. On day 3 after surgery, the dog was able to eat small amounts of food. The chest tube was removed due to minimal fluid aspiration (11 mL of hemorrhagic fluid) and no air aspirated in a period of 24 h. On day 4 after surgery, the dog was discharged after a total of 8 days of hospitalization.

Ten days after the surgery, the dog presented for a re-check examination. The owner reported that the dog maintained a normal energy and appetite. There was a 24-h period of persistent wet, productive cough the day that the dog was discharged that changed to a dry, hacking cough that persisted for the remainder of his time at home. He had intermittent bilateral serous nasal discharge as well. There was a 7-days period of diarrhea that resolved with a bland diet. Thoracic radiographs were performed to recheck pneumonia. There was improving bronchopneumonia with improvement of patchy unstructured interstitial pulmonary pattern coalescing to alveolar pulmonary pattern in the caudal subsegment of the left cranial lung lobe. The pneumothorax resolved (Figure 3).

Five weeks after surgery, thoracic radiographs were repeated. The interpretation indicated complete resolution of pneumonia. At that point the chronic regurgitation was addressed. A swallow study was performed due to concern of regurgitation as the cause for initial development of pneumonia. This was interpreted as normal. A gastrointestinal panel was performed that assessed cobalamin, folate, pancreatic lipase immunoreactivity, and trypsin-like immunoreactivity. Serum folate was decreased below reference interval. This was interpreted as a malabsorptive process. The remainder of the values were unremarkable. The dog was started on a diet trial for 4–6 weeks of a hydrolyzed diet and oral folate supplementation (11.7 mcg/kg PO q24). To address the chronic regurgitation, cisapride (0.44 mg/kg PO q8) was continued up to date of this publication.

Up to the point of this case-report, the dog is doing well at home. The owner reports that the coughing has resolved. The dog occasionally sneezes and produces bilateral serous nasal discharge that resolves without intervention.

## Discussion

This report presents the diagnosis, medical and surgical treatment, postoperative care, and recheck status of a dog with necrotizing *E. coli* pneumonia leading to a pneumothorax. Furthermore, this report provides information on the management of a necrotizing pneumonia case and demonstrates a positive outcome based on resolution of pneumonia and pneumothorax.

There are several limitations to the management of this case. First, we did not submit a culture of the thoracic fluid obtained from the chest tube. Based on cytology, however, the bacteria observed appeared morphologically similar to those seen in the initial bronchoalveolar lavage, and we therefore assumed the same infectious agent was present. Second, we did not specifically request culture for slow-growing filamentous bacteria such as *Nocardia* and *Actinomyces*. These organisms can cause severe pneumonia in dogs and cats, and their diagnosis is often challenging. Although *Nocardia* and *Actinomyces* have been reported as causes of necrotizing pneumonia in humans (10, 11) and occasionally in animals (12), their culture is difficult, frequently requiring specialized media and prolonged incubation periods. Even with appropriate culture technique, negative results do not exclude infection, as false negatives are common due to the slow-growing nature of these organisms. Therefore, not specifically investigating for filamentous bacteria represents a limitation of our case management.

The dog was continued on the same antibiotic, enrofloxacin, based on initial culture results from the BAL. This bacterial infection likely did not respond to initial antibiotic trials since our culture demonstrated resistance to doxycycline and amoxicillin-clavulanic acid (Table 2). Further investigation including culture for *Nocardia* and *Actinomyces* was not performed, and their possible contribution to the chronicity and severity of the pneumonia cannot be excluded.

Necrotizing pneumonia is a complication of a severe parenchymal infection. In humans, necrotizing pneumonia is generally secondary to an infection that progresses to pulmonary necrosis and lies on a spectrum in between pulmonary abscess and pulmonary gangrene (5). This is characterized by pulmonary inflammation with consolidation, cavitation and peripheral necrosis. Development of this condition occurs when severe inflammation hinders the amount of blood flow to the alveolar capillaries leading to devitalization of lung parenchyma (5). Lack of blood supply prevents adequate perfusion of antibiotics; therefore, causing an uncontrolled infection and further destruction of tissue (1). We believe this was the case in this dog, as clinical deterioration occurred despite appropriate antibiotic treatment. Aggressive, prolonged antibiotic therapy is the first-line treatment in human medicine. Indications to escalate to surgical treatment are not well defined but include persistent fever with leukocytosis, empyema, bronchopleural fistula with hemoptysis, and impaired respiratory function caused by necrotic parenchyma (5, 13). As the disease progresses, empyema, pleural effusion and pneumothorax can develop as well as pulmonary gangrene (1).

The development of necrotizing pneumonia is more so described in human literature, particularly in pediatrics. It is considered a community-acquired pneumonia (CAP) with necrotizing pneumonia occurring at a rate of 3.7% of all CAP cases (14). In humans, *S. aureus* and *Streptococcus* spp. are the most common causative agents, however, there are many other documented infectious agents including *B. dermatitidis*, *M. pneumoniae*, H1N1 influenza, *P. aeruginosa*, *Klebsiella* spp., and *E. coli* (2–5). Many cases occurring in children happen in previously healthy individuals whereas adults are generally immunocompromised. The presenting clinical signs are those associated with pneumonia: hyperthermia, chest pains, tachypnea, and coughing. When clinically affected, adults and children remain febrile and show signs of respiratory distress despite adequate antibiotic therapy. Sequelae of pneumonia includes pleural effusion, pyopneumothorax, and empyema (15). Diagnostics including CT scans, blood and pleural fluid cultures are routinely performed. Treatment requires prolonged hospitalization and administration of antibiotics which are continued until 4 weeks after a negative blood culture; however, treatment can escalate to thoracic drain placement, surgical intervention, and mechanical ventilation (15). In children and adults, the clinical outcome varies depending on severity of lung necrosis which ranges from mild to life-threatening. Death is uncommon in children with many of these patients’ experiencing resolution of clinical signs and radiographic recovery in 5–6 months; whereas adults have worse prognosis with an estimated 40–50% mortality rate (5).

Aspiration pneumonia is the cause in 5–15% of all pneumonia cases in human medicine (16). In dogs, there is an incidence of development of aspiration pneumonia post-operatively at 0.17% (17); however, there is not a general statistic that includes all causes of aspiration pneumonia known to the author. There are many causes for aspiration of gastric contents including gastrointestinal disease (60%), esophageal disease (38%), neurologic disease (12%), laryngeal disease (10%), and recent anesthetic events (7%) (18). In this dog, we suspect the primary mechanism of aspiration is chronic regurgitation with an unknown etiology. This dog’s causative agent, *E. coli*, is a common pathogen in veterinary aspiration pneumonia (19). In dogs with uncomplicated aspiration pneumonia, the survival rate greatly varies from 30 to 80% (20). We believe that this dog was exposed to *E. coli* secondary to regurgitation and aspiration of gastric contents although other causes and precipitating factors such as a viral component, *Nocardia*, and *Actinomyces* cannot be ruled out. Further assessment for more difficult to culture species of *Nocardia* and *Actinomyces* was not performed, even though these are clinically important pathogens to the development of necrotizing pneumonia (citation). Even with appropriate culture technique, these bacterial species are difficult to culture and negative results can be false; however, it is a limitation of the case that the filamentous bacteria were not investigated further.

*Escherichia coli* was cultured via bronchoalveolar lavage during the second day of hospitalization. After surgery, culture of the affected lung parenchyma was negative. This is most likely due to administration of intravenous antibiotics for 4 days prior. The use of antibiotics, whether empirical or targeted, decreases the probability of obtaining positive culture results with biopsies (21). It is possible that the bacterial component of disease was controlled with enrofloxacin, but the sequelae—including the necrotized tissue and pneumothorax—could not be resolved without surgery. Antibiotics were continued despite a negative lung tissue culture, based on the initial sensibility antibiogram, until radiographic resolution of pneumonia. The antibiotic was maintained because the dog continued to improve clinically following removal of necrotic lung tissue.

There are several classifications of pneumothorax including traumatic and atraumatic. Atraumatic pneumothorax can be further characterized as primary or secondary. This dog is classified as a secondary spontaneous pneumothorax as this developed due to an underlying pulmonary disease (22). Indications for surgical intervention in relation to a pneumothorax in human medicine include a continuous air leak for greater than 7 days, recurrent unilateral pneumothorax and bilateral pneumothorax (22). In veterinary medicine, surgery is the treatment of choice for dogs with a spontaneous pneumothorax that do not have an identified non-surgical disease or diffuse disease (23). Dogs that receive solely medical management have high recurrence and mortality rates of 50 and 53%, respectively (23). It should be noted that none of the dogs in this study developed a spontaneous pneumothorax secondary to necrotizing pneumonia. Although the recommendation is to perform surgery in these cases, there is no consensus on specific indications in veterinary medicine for surgical management. Regarding the dog in this case report, surgical management was elected based on a financial discussion, lack of improvement of the condition, and lack of evidence for diffuse disease.

## Concluding remarks

This case report describes the successful management of necrotizing *E. coli* pneumonia complicated by a subsequent pneumothorax. *E. coli* was initially cultured from a bronchoalveolar lavage sample, and the dog was treated with intravenous enrofloxacin based on sensitivity results. Surgical intervention was required due to the persistence of the pneumothorax. To our knowledge, this is the first veterinary report describing clinical and radiographic resolution of necrotizing *E. coli* pneumonia, a condition with scarce published data and uncertain prognosis. This case demonstrates that necrotizing pneumonia can be successfully managed with appropriate escalation of treatment modalities and may carry a favorable outcome in dogs. Clinicians should remain vigilant for the potential development of pneumothorax in severe cases.

## Data Availability

The original contributions presented in the study are included in the article/Supplementary material, further inquiries can be directed to the corresponding authors.
